# Translational regulation of human papillomavirus mRNAs in carcinogenesis: old questions and new insights

**DOI:** 10.3389/fcell.2025.1676001

**Published:** 2025-10-07

**Authors:** Noemi Baranda-Ávila, Giovanna Maldonado, Dora E. Vélez, Greco Hernández

**Affiliations:** ^1^ mRNA and Cancer Laboratory, Unit of Biomedical Research on Cancer, Instituto Nacional de Cancerología (INCan; National Institute of Cancer), Mexico City, Mexico; ^2^ Tecnologico de Monterrey, Escuela de Medicina y Ciencias de la Salud, Mexico City, Mexico

**Keywords:** papillomavirus, cervical cancer, translational control, eIF4E, codon usage, untranslated region

## Abstract

Persistent infection with high-risk human papillomaviruses (HR-HPVs) is a major etiological factor in the development of cervical cancer, which ranks as the fourth leading cause of cancer-related mortality among women worldwide. HR HPV types 16 and 18 cause more than 70% of all cases. These viruses encode the proteins E1, E2, E1^E4, E4, E5, E6, E7, L1, and L2, through a complex array of polycistronic mRNAs. For decades, research on HPV gene expression has focused predominantly on transcriptional activity and mRNA splicing. In contrast, the mechanisms underlying the translation of mRNAs remain poorly understood. Whereas the translational regulation of E1, E2, E6, and E7 has been elucidated, the translation mechanisms for E5, L1, and, L2 proteins are still unclear. We hypothesized that their translation may occur via internal ribosome entry sites (IRESs). Other critical questions also remain open, including how the viral-cellular chimeric transcripts generated upon virus genome integration into the host DNA are translated, as well as how the translation of polycistronic viral mRNAs is regulated during the differentiation of epithelial cells—a process that is central to HPV-induced carcinogenesis. This review summarizes current knowledge showing that the translation of HPVs mRNAs is subjected to tight regulation, highlights unresolved questions, and discusses potential therapeutic implications of targeting the translational machinery in HPV-related cancers.

## Introduction

Cervical cancer ranks fourth in incidence and third in mortality among neoplasias affecting women worldwide. Its burden might reach highest mortality rates in more than 40 low-income countries (National Cancer Institute, 2021; LaVigne et al., 2017; Sung et al., 2021; WHO, 2022). HPV prevalence is markedly higher in emerging regions, reaching 42.2% compared to 22.6% in high-income regions (Pavelescu et al., 2025). Persistent infection of the genital tract with high-risk (HR) human papillomavirus (HPV) is the primary cause of most cervical cancer cases. Among these, HR-HPV types 16 and 18 are responsible for over 70% of cases. Moreover, about 90% of anal cancer, 75% of vaginal cancer, 70% of oropharyngeal and vulvar cancers, and 63% of penile cancers are caused by HR-HPVs across the globe (Alteri et al., 2025). Effective vaccines against HR-HPV have been developed, and HPV vaccination was introduced in 73 countries during 2010–2019 and in 30 additional countries during 2020–2023. Global first-dose HPV coverage among girls increased from 17% in 2019 to 27% in 2023, and full-course coverage rose from 13% in 2019 to 20% in 2023 (Jones et al., 2024). According to World Health Organization, global first-dose coverage further increased to 31% in 2024. However, this is still far below the 2030 target of 90% coverage because their use remains limited or non-existent in many countries (Collaborators, 2025), highlighting cervical cancer as a major public health challenge worldwide.

HR-HPVs infect basal keratinocytes of the mucosal epithelium, leading to the formation of low-grade intraepithelial lesions. Once within the host cells, the viral genome replicates as an extrachromosomal DNA inside the cell nucleus. As epithelial cells divide and differentiate, HPV gene expression couples with cell differentiation, and cells progress to productive viral states, leading to cell proliferation, immortalization, and the development of malignant phenotypes (Nelson and Mirabello, 2023; Obanya et al., 2025; Pavelescu et al., 2025; Rosendo-Chalma et al., 2024). This process has been extensively studied with regard to the transcription and splicing of viral mRNAs (Doomer et al., 2024; Olmedo-Nieva et al., 2018; Wang et al., 2025; Yu et al., 2022) (https://pave.niaid.nih.gov/#explore/transcript_maps). However, the translation of viral mRNAs remains the least studied aspect of viral gene expression (Kajitani and Schwartz, 2022).

Notably, translational control is emerging as a key process of viral proteins synthesis, opening novel avenues to target the translational machinery to combat HVP-induced cervical cancer. We review the old, yet unresolved questions regarding the translation of viral mRNAs, alongside recent advances in our understanding of viral protein synthesis regulation and their implications for the design of new therapeutic strategies.

### Translation in eukaryotes

Translation, i.e., the decoding of genetic information of a mRNA into peptide by the ribosome and translation factors using a genetic code, largely determines in all cells protein abundance and the proteome composition in normal physiology, stresses and diseases (Hernández and Tettweiler, 2012). Accordingly, a myriad of mechanisms to regulate mRNA translation exists in cells and virus (Hernández et al., 2020; Hershey et al., 2019; Jan et al., 2016; Kwan and Thompson, 2019). Translation consists of four main stages, namely initiation, elongation, and termination. In some physiological situations a fourth stage may happen for some mRNAs, i.e., ribosome recycling. However, the global process of translation is largely controlled at the initiation step (Hershey et al., 2019). Different mechanism account for the initiation step, namely the canonical mechanism and the non-canonical leaky scanning, ribosome shunting, termination-reinitiation, and IRES-dependent mechanisms.

### The canonical mechanism to initiate translation

**FIGURE 1:**
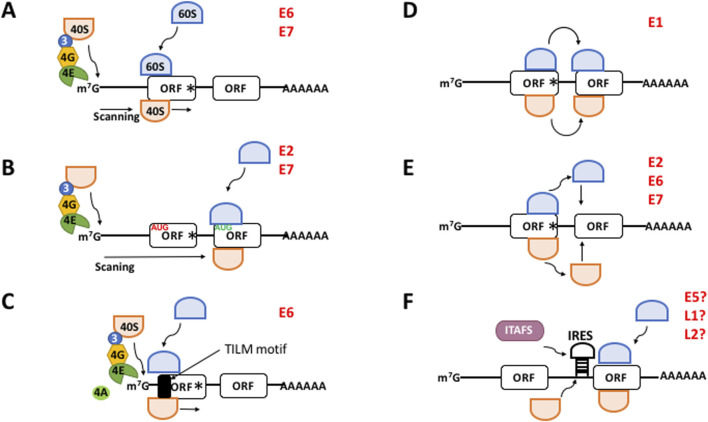
The different mechanisms to initiate translation in eukaryotes. **(A)** Canonical, cap-, eIF4E-, and scanning dependent mechanism. **(B–F)** Non-canonical mechanisms. **(B)** Leaky scanning. mRNAs containing a weak Kozak AUG contexts (in *red*) allow the ribosomal 40S subunit to skip the first ORF and recognize the downstream AUG (in *green*) of the next ORF which is in good context. **(C)** Translation Initiation of Leaderless mRNAs (TILM)-dependent mechanism. Extremely short 5′-UTR and a TILM motif within the E6 ORF promote translation initiation. **(D)** Ribosome shunting. The 40S ribosome shunts over a transcript segment landing downstream to reach the AUG codon of the next ORF. **(E)** Termination-reinitiation. After translation has terminated ribosomes quit the mRNA. Then a new ternary complex is loaded onto the 40S ribosome and it resumes scanning to start translation of the next ORF. **(F)** IRES-dependent mechanism. The 40S ribosome directly binds an IRES next to the AUG start codon to initiate translation. This process may need the help of *IRES*-trans acting factors (*ITAFS*). *m* ^*7*^ *G*, methyl-7 GTP cap structure of mRNA; *40S*, ribosomal subunit 40S; *60S*, ribosomal subunit 60S; *3*, eIF3; *4G*, eIF4G; *4E*, eIF4E; *Black box*, translation initiation of leaderless mRNAs (*TILM*) motif; *ORF*, open reading frame; *AAAA*, poly(A) tail; *IRES*, internal ribosome entry site. The viral proteins with a proposed mechanism are indicated in *red*. Hypothetical IRES-driven translation for E5, L1, and L2 is indicated with a?

Most eukaryotic mRNAs initiate translation in the canonic manner, termed cap-dependent mechanism. It begins with the mRNA recruitment via recognition of the cap structure (7-methyl guanosine) at the 5′ of the mRNA by eukaryotic initiation factor (eIF) 4E. During this process, eIF4G binds eIF4E, eIF4A and poly(A)-binding protein (PABP). Subsequently, a free 40S ribosomal subunit in complex with eIF1, eIF1A, eIF3, eIF5 and a ternary complex (TC, consisting of eIF2 bound to an initiator Met-tRNA_i_^Met^ and GTP) forms a 43S pre-initiation complex (PIC). eIF4G further interacts with the ribosome-bound eIF3 to promote recruitment of the 43S PIC to the mRNA 5′-untranslated region (5′-UTR) (Brito Querido et al., 2024; Pelletier and Sonenberg, 2019). After the mRNA is recruited, the 43S PIC scans the 5′-UTR to reach the translation initiation site (TIS), most frequently an AUG codon (Figure 1A) (Brito Querido et al., 2024; Kwan and Thompson, 2019; Pelletier and Sonenberg, 2019). A context sequence surrounding the TIS, termed the “Kozak motif”, is required for optimal AUG codon recognition by the ribosomal complex. For human mRNAs, this motif consists of the sequence GCCA/GCCAUGG (Hernández et al., 2019). AUG recognition triggers 60S ribosomal subunit joining to assemble an 80S initiation complex which enters in successive rounds of peptide elongation (Brito Querido et al., 2024; Kwan and Thompson, 2019; Pelletier and Sonenberg, 2019). When a stop codon is recognized in the A site of the ribosome eukaryotic releasing factor (eRF) 1 promotes peptide liberation.

### Non-canonical translation initiation mechanisms

In addition to the canonical pathway, translation initiation may happen via other mechanisms, which mostly account for the translation of internal ORF within polycistronic mRNAs in many viruses.

In the “leaky scanning” (Hinnebusch et al., 2004), mRNAs containing an unfavorable Kozak (i.e., “weak”) motif allow the ribosomal 43S PIC skip the first open reading frame (ORF) and recognize a downstream ORF AUG (Figure 1B). Recently, we demonstrated that E6,E7 mRNAs from HPV-18 possesses extremely short 5′-UTRs (0–5 nucleotides) in cervical tumors which drive the translation of E6 (García et al., 2021), conforming a novel non-canonical mechanism of translation initiation (Figure 1C). In this virus, E6 is translated in a scanning-independent manner but is dependent on the mRNA cap and the poly(A) tail, eIF4E, eIF4A, and on a novel RNA motif within the coding region of the E6 cistron that we termed TILM (for Translation Initiation of Leaderless mRNAs). Thus, in the absence of a 5′-UTR, TILM controls the translation of E6 (García et al., 2021). In the “ribosome shunting” (Kwan and Thompson, 2019), specific *cis*-elements within the mRNA promote that the 43S PIC bypasses or shunts over a segment of the transcript, lands downstream and resumes scanning to reach the TIS (Figure 1D). In the “termination-reinitiation” (Jackson et al., 2012), after translation has terminated, a new TC is loaded onto a 40S subunit and it resumes scanning to start the translation of a downstream ORF on the same mRNA (Figure 1E). For some other transcripts, translation initiation is driven by an internal ribosome entry site (IRES) (Kwan and Thompson, 2019), a *cis*-located mRNA region that promotes ribosomal recruitment just nearby the TIS in a cap- and eIF4E-independent manner and without scanning. This process may be promoted by the activity of cellular IRES-*trans*-acting factors (ITAFs) (Figure 1F).

### Function of the viral proteins during infection and carcinogenesis

HPVs encode the early proteins E1, E2, E4, E5, E6, and E7 from the early (E) control region of the viral genome, and the capsid proteins L1 and L2 from the long control region (LCR) (Doorbar et al., 2015; Nelson and Mirabello, 2023; Rosendo-Chalma et al., 2024). E1 is a helicase that, in cooperation with E2, drives viral genome replication. E2 also regulates the viral cycle life and controls the transcription of the E6 and E7 oncogenes. E4 plays a role in virion assembly and facilitates the release of newly formed viral particles. Collectively, E1, E2, and E4 drive integration, replication, and transcription of the viral genome (Bhattacharrjee et al., 2021; Della Fera et al., 2021; Evande et al., 2023). E5 is involved in membrane signaling, cell proliferation, apoptosis, oncogenesis, and angiogenesis (Basukala and Banks, 2021; Estêvão et al., 2019). E6 and E7 are potent oncogenic proteins that interact with a wide array of cellular proteins. Their activity is essential for the development and maintenance of the malignant phenotype. Impairment of these proteins, or the inhibition of their gene expression, induces cellular senescence and growth arrest in HPV-positive cancer cells (Basukala and Banks, 2021; Estêvão et al., 2019). The principal function of E6 is its interaction with the tumor suppressor p53 (Martinez-Zapien et al., 2016). This interaction inhibits apoptosis, thereby allowing cells harboring genomic damage to evade cell death and continue proliferating. In addition, E6 engages with other host proteins, contributing to carcinogenic phenotypes, cellular immortalization, and cell invasion (Panczyszyn et al., 2018). E7 promotes degradation of the tumor suppressor retinoblastoma (pRB), leading abnormal centrosome synthesis and aneuploidy, ultimately resulting in genomic instability (Duensing et al., 2001). In addition, E7 interacts with other cellular targets to promote deregulation of cell proliferation and increased invasive potential. E6 and E7, acting independently or in concert, modulate a wide range of cellular pathways critical for cancer progression. These include immune evasion, dysregulation of cellular energetics, inhibition of growth suppressor pathways, sustained proliferative signaling, epigenetic reprogramming, metastasis, tumor-associated inflammation, and angiogenesis (Basukala and Banks, 2021; Estêvão et al., 2019).

L1 and L2 are structural proteins that constitute the capsomers of the viral capsid. Indeed, current vaccines against HPV are designed to elicit immune responses primarily targeting the strong antigenicity of L1 (Tsakogiiannis et al., 2022). In addition, the L1 gene sequence is used for HPV genotyping, while L2 plays a role in the intracellular transport of viral DNA to the nucleus of infected cells (Doorbar et al., 2015; Graham, 2017; Rosendo-Chalma et al., 2024). In the following sections, we review the mechanisms by which viral proteins are synthesized from a myriad of polycistronic mRNAs.

### HR-HPVs proteins are synthesized using different mechanisms to initiate translation

**FIGURE 2:**
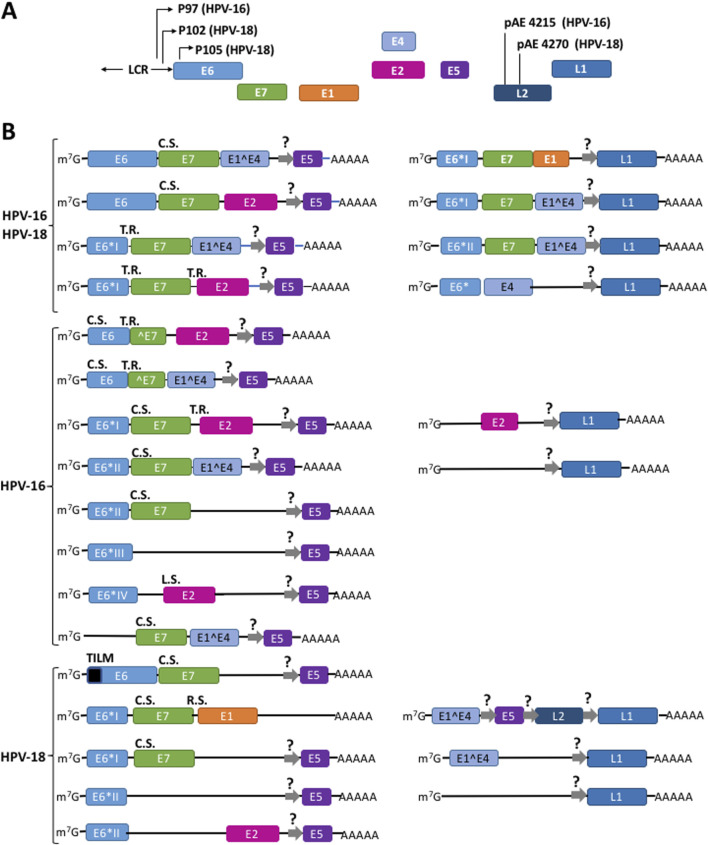
Different mechanisms drive the translation of a plethora of polycistronic mRNAs to synthesize the viral HPV proteins. **(A)** Structure of HR-HPV 16 and 18 genomic DNA. **(B)** Some mRNAs originated from episomal genome and from viral DNA integrated into the cellular genome. *Thin arrows* represent the promoters *P*_*97*_ for *HPV-16* and *P*_*102*_ and *P*_*105*_ for *HPV-18*; *pAE*, early polyadenylation signals; *LCR,* long control region; *Squares* represent exons; *lines* into mRNAs represent intercistronic regions. *R.S.*, ribosome shunting; *T.R*., termination-reinitiation; *L.S.*, leaky scanning; *C.S.*, canonical scanning. E5, L1, and L2 cistrons might be translated from hypothetical IRES (thick *gray arrows* with a question mark). Adapted from Yu et al. (2022), Wang et al. (2025), García et al. (2024), Wang et al. (2025), Yu et al. (2022). A fuller list of the HPVs transcripts is described in the Papilloma Virus Episteme (PaVE) (Doomer et al., 2024; Van Doorslaer et al., 2017) at https://pave.niaid.nih.gov/#explore/transcript_maps and references therein.

HR-HPV proteins are expressed by polycistronic mRNAs transcribed from the different promoters in the long control region (LCR), the early (E) region and the late (L) region of the HPV-16 and HPV-18 genomes, respectively (Figure 2A) (Doorbar et al., 2015; Graham, 2017; Rosendo-Chalma et al., 2024). Overall, the high complexity of alternative splicing events of viral transcripts produces a plethora of mRNAs with diverse structures. In all mRNAs, the first ORF encodes E6. Full-length E6 is only translated from unspliced transcripts (De la Cruz-Hernández et al., 2005; Mesplede et al., 2012; Moral-Hernández et al., 2010; Schneider-Gädicke and Schwarz, 1986; Smotkin and Wettstein, 1986; Stacey et al., 1995; Toots et al., 2014). In addition, shorter versions of E6 termed E6*I, E6*II, E6*III, E6*IV, E6*V, E6*VI, E6^E7, E6^E7*I, and E6^E7*II are also expressed from alternative splicing events of the same mRNA (Ajiro and Zheng, 2015; Brant et al., 2018; Olmedo-Nieva et al., 2018). E7 is synthesized from several spliced transcripts and from unspliced mRNAs (Doorbar et al., 1990; Durst et al., 1991; Mesplede et al., 2012; Moral-Hernández et al., 2010; Sherman et al., 1992; Smotkin et al., 1989; Tang et al., 2006; Thierry et al., 1987). Moreover, multiple alternatively spliced transcripts and clusters of heterogeneous mRNAs expressed from different transcription start sites have also been identified in cultured cells, raff cultures, and tumorigenic tissues that only contain either E6 or E7 as the sole cistron (Braunstein et al., 1999; Glahder et al., 2003; Grassmann et al., 1996; Rosenstierne et al., 2003; Schneider-Gädicke and Schwarz, 1986; Smotkin and Wettstein, 1986). Figure 2B shows most of the multiple, polycistronic mRNAs transcribed from both the episomal and the integrated HR-HPVs genome (Graham, 2010; Graham, 2017; Wang et al., 2025).

Surprisingly, In cultured cells and epithelial rafts the 5′-UTR regions of E6,E7 mRNAs are 7-9 nucleotides in length for HPV-16 (Stacey et al., 2000), and in HPV-18 the most represented transcripts possess either 3 nucleotides long 5′-UTR or completely lack it, i.e., the transcription starts at the A of the AUG codon that initiates translation (García et al., 2021; Romanczuk et al., 1990; Schneider-Gädicke and Schwarz, 1986; Smotkin and Wettstein, 1986; Thierry et al., 1987; Wang et al., 2011). Thus, the structure of viral mRNAs contrasts drastically with that of mRNAs across eukaryotes (both cellular and viral), with a 5′-UTR length average of 53–164 nucleotides (Leppek et al., 2018; Ryczek et al., 2023). In multicellular species, short 5′-UTRs have only been found in the human nuclear-encoded mRNAs harboring the element Translation Initiation of Short 5′-UTR (TISU), consisting of 12–30 nucleotide-long 5′-UTRs (Elfakess and Dikstein, 2008; Elfakess et al., 2011), and some mitochondrial mRNAs with 5′-UTRs shorter than five nucleotides (Gandin et al., 2016). Recently, we demonstrated that in cervical tumors E6,E7 mRNAs from HR HPV-18, HPV-39 and HPV-45 possess 5′-UTRs of 0–5 nucleotides which drive the translation of full-length E6 (García et al., 2021). For HPV-18, E6 is translated in a scanning-independent manner and is dependent on the RNA motif within the E6 coding region termed TILM, that drives the translation of E6 (García et al., 2021). However, the proteins that regulate E6 synthesis via the TILM motif *in vivo* are unknown. In contrast, the HPV-16 E6 cistron with a 7-9 nucleotides-long 5′-UTR is translated by the canonical mechanism (Stacey et al., 2000).

The synthesis of polycistronic mRNAs raises the fundamental question of how E1, E2, E4, E5, and E7 are translated downstream of E6. Evidence from cervical cancer cell lines suggest that the HPV-16 and HPV-18 E7 cistron is translated from spliced E6*I mRNA via translation termination-reinitiation (Tang et al., 2006). In a cell-free coupled *in vitro* transcription/translation system using rabbit reticulocyte lysates and a synthetic HPV-16 E6,E7 bicistronic mRNA, Tan et al. showed that full-length E6 translation also promotes termination-reinitiation on the E7 cistron AUG 3 to 6 nucleotides downstream the E6 stop codon (Tan et al., 1994).

The translation HPV-18 E1 has been studied by plasmid transient transfections of the green monkey kidney COS-7 fibroblast-like cultured cells (Remm et al., 1999). The distance between the E7 and E1 ORF consists of 6 nucleotides, and Remm et al. (1999) demonstrated that the translation of the E1 cistron downstream of E7 occurs by ribosome shunting. This group demonstrated that the translation of the E1 ORF is inhibited from monocistronic transcript that might arise from spurious transcription sites or partial degradation, and its translation is strongly favored from polycistronic rather than monocistronic mRNAs as a means to ensure E1 premature synthesis in early stages of the viral infection cycle.

The translation of HPV-16 E2 ORF has been studied in both COS cells and cell-free *in vitro* translation systems. In the transcripts E6*I,E7,E2,E5, the E7 stop codon and the E2 AUG are separated by 33 nucleotides and the termination-reinitiation mechanism could translate E2 after E7 is translated. On the other hand, in the E6*IV transcript, the E2 AUG is out of frame and upstream of the E6*IV termination codon. Moreover, E2 AUG has a stronger Kozak motif than E6*VI AUG. Thus, avoiding the upstream E6*IV AUG leaky scanning could also be the mechanism to initiate E2 translation (Alloul and Sherman, 1999).

HPV-16 E5 translation has been studied only in the mouse fibroblast NIH3T3 cells (Johnsen et al., 1995). HPV-16 and HPV-18 E1^E4, E5 mRNA is the most abundant transcript found in all cervical neoplasias (Sherman and Alloul, 1992; Stoler et al., 1992). However, HPV-16 E5 is not translated from this mRNA because it is rich in AUG triplets upstream the initiator AUG codon. In contrast, E2 and E5 are well expressed from the E2,E5 transcripts lacking the upstream E^E4 cistron, suggesting a synchronized, positive translational regulation of both E2,E5 ORFs (Johnsen et al., 1995). However, the mechanisms of E5 translation has not been elucidated. We have proposed the possible existence of IRES to drive the expression of E5 ORF (García et al., 2024) (represented in Figure 2B by gray arrows). However, experimental analyses should be performed to prove it.

HPV-18 E7 in COS transfected cells E7 is translated by the canonical mechanism that might result from the translation of a subset of short mRNAs originated from the P_105_ early promoter lacking the first nucleotides of E6 ORF (Remm et al., 1999). HPV-16 E7 translation was also studied in cell-free rabbit *in vitro* translation lysates and plasmid transfection of HeLa cells (Stacey et al., 1995; Stacey et al., 2000). In contrast, it was found that E7 ORF is translated by an exceptionally pervasive leaky scanning process in which the ribosomal initiation complex does not recognize the AUG start codon of the upstream E6 cistron, and scans through the mRNA over 13 upstream AUG codons until it reaches the E7 ORF TIS (Stacey et al., 1995; Stacey et al., 2000).

L1 and L2 are transcribed from the late region of the HR-HPVs genome (Figure 2C) (Doorbar et al., 2015; Graham, 2017; Nelson and Mirabello, 2023; Rosendo-Chalma et al., 2024). So far, no mechanism of mRNA translation has been elucidated for the L1 and L2 mRNAs, but we propose here that they might be translated by IRESs. Further experimental analyses also should be performed to prove it. In Table 1 we summarize the mechanisms used to translate the different HPV-16 and HPV-18 cistrons.

**TABLE 1:** Mechanism of translation used to translate the viral cistrons.

Virus	Cistron	Mechanism	Reference
VPH-16	E1	Not known	
	E1^E4	Not known; possible IRES	This review
	E2	Termination-reinitiation or leaky scanning	Alloul and Sherman (1999)
	E5	Not known; possible IRES	García et al. (2024)
	E6	Canonical	Stacey et al. (2000)
	E7	Termination-reinitiation or leaky scanning	Stacey et al. (1995), Stacey et al. (2000), Tan et al. (1994), Tang et al. (2006)
	L1	Not known; possible IRES	This review
	L2	Not known	
VPH-18	E1	Ribosome shunting	Remm et al. (1999)
	E1^E4	Not known; possible IRES	This review
	E2	Not known	
	E5	Not known; possible IRES	García et al. (2024)
	E6	Scanning-independent using extremely short (0–3 nucleotides long) 5′-UTRs and an RNA sequence within the ORF; it also requires the mRNA cap and poly(A) tail; it requires eIF4E and eIF4A	García et al. (2021)
	E7	Termination-reinitiation or canonical	Remm et al. (1999), Tang et al. (2006)
	L1	Not known; possible IRES	This review
	L2	Not known; possible IRES	This review

### Codon usage regulates viral mRNA translation

In all organisms, most amino acids are encoded by multiple synonymous codons. However, codon usage is not uniform across the different species. The preference for specific codons over others during mRNA translation is referred to as codon bias or codon preference (Parvathy et al., 2022). Due to the absence of their own translational machinery, viruses have evolved to optimize their codon usage to match that of their host. By aligning their codon usage patterns with the host’s tRNA pool, viruses enhance the translational efficiency of their own proteins (Kaleem et al., 2025; Simón et al., 2021).

Viruses often employ suboptimal codon usage to reduce the translation rate of viral proteins as a strategy to evade host immune surveillance and response. This involves the use of codons that are less favorable for translation (Kaleem et al., 2025; Mordstein et al., 2021). Indeed, in a wide range of HPV types, codon bias shows poor alignment with the average human codon usage preferences (Cladel et al., 2010; Félez-Sánchez et al., 2015; Ren et al., 2025; Zhao and Chen, 2011). Moreover, a study of the codon usage bias across 79 HPVs showed strong differences among the eight cistrons encoding viral proteins toward 18 codons with T at the third position. Among them, L2 ORFs showed the highest codon bias and E4 the lowest (Zhao et al., 2003). The codon bias of E6,E7 mRNAs poorly matches the preferred codons by host cells, which strongly diminishes the rate of protein translation. As expected, changing the rare toward optimal codons of human cells (Müller, 2005) or the co-transfection of different cultured cells with a plasmid expressing tRNA^Ser(CGA)^ (Gu et al., 2004) enhanced protein synthesis of the viral proteins. Transfection of a codon-optimized HPV-16 E6 gene also increased protein expression in cultured human cell (Lin et al., 2006; Samorski et al., 2006). Regarding E7, supplementation of rat liver tRNAs to the pool of a cell-free *in vitro* translation system from rabbit reticulocyte (De Pasquale and Kanduc, 1998), or the use of a codon-optimized plasmid transfected into human cells (Steinberg et al., 2005) significantly enhanced E7 synthesis. It was further observed that the codon bias of HPV genes couples viral gene expression with epithelium differentiation throughout virus life cycle and carcinogenesis (Gu et al., 2007; Zhao et al., 2005; Zhou et al., 1999).

The study of codon usage bias has been fundamental to the design of vaccines against HR-HPVs. In the two widely used commercial vaccines, Gardasil and Cervarix, codon optimization was employed to enhance the production of virus-like particles (VLPs) derived from the L1 protein, which serves as the primary immunogenic target (Kaleem et al., 2025; Markowitz and Schiller, 2021).

### Integrated HPVs produce chimeric E6, E7 mRNAs fused with cellular genes

The life-cycle of mRNAs is tightly controlled by numerous *cis*-regulatory elements located at the 5′- and 3′-UTRs that control the translation and transport of the transcripts, are target of nucleases, or bind to a myriad of regulatory molecules (Leppek et al., 2018; Mayr, 2017; Ryczek et al., 2023). Consequently, mutations in the mRNA UTRs may lead to the uncontrolled expression of a wide variety of mRNAs and, often trigger the development of diverse diseases (Chatterjee and Pal, 2009), including cancer (Chan et al., 2023; Huang et al., 2022; Li and Lu, 2013; Schuster and Hsieh, 2019; Silanes et al., 2007; Zhang et al., 2022).

**FIGURE 3:**
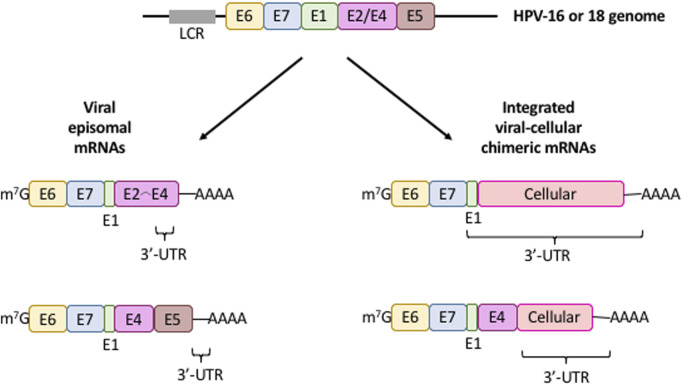
mRNA expression patterns from integrated (fusion) and episomal HPV 16/18 DNA. Diagram illustrating the characteristic structure of viral mRNAs produced from intact episomal genomes (*left*) or the viral DNA integration into the cellular genome (*right*). Only the cistrons (*colored boxes*) controlled by the long control region (*LCR, gray box*) is depicted. The 3′-UTR of these transcripts is shown. *Colored boxes*, viral cistrons; *m* ^*7*^ *G*, mRNA cap structure; *AAAA*, mRNa poly(A) tail.

The regulatory elements that control polyadenylation, degradation, localization, and transport of mRNAs are mostly located in the 3′-UTR (Mayr, 2017). In most tumors, HPV DNA is generally integrated into the genome of epithelial cells, unlike productive infections where is found in episomal form (Figure 3). The viral genome integration is a stochastic process over almost all chromosomes. During this, some viral genomes may be integrated into cancer-related genes which may promote tumorigenesis. Moreover, as a consequence of this integration, the *E6* and *E7* genes lose their natural 3′-UTR, disrupt the viral E2 ORF, and gain a cellular DNA fragment that is transcribed as a viral-cellular chimeric 3′-UTR (Figure 3). The promoter controlling E6 and E7 genes contains binding sites for E2 protein and binding of E2 represses E6, E7 mRNA transcription. Therefore, loss of the E2 cistron leads to an increase in the E6, E7 mRNA transcription and protein synthesis (Jeon et al., 1995; Lace et al., 2011; Vinther et al., 2005; Wentzensen et al., 2002; Wentzensen et al., 2004). Thus, the main consequence of HPV genome integration is the upregulation and stabilization of E6,E7 mRNAs, leading to the E6 and E7 overexpression and the promotion of carcinogenesis (Couturier et al., 1991; Durst et al., 1987; Ehrig et al., 2020; Jeon and Lambert, 1995; Wentzensen et al., 2002; Ziegert et al., 2003).

The viral genome integration into the cell genome plays a critical role in triggering carcinogenesis and is one of the molecular features that best defines the transition to carcinogenic transformation (Groves and Coleman, 2015; Groves and Coleman, 2018; Wentzensen et al., 2004). Although the stability of chimeric mRNAs increases (Ehrig et al., 2020; Jeon and Lambert, 1995; Vinther et al., 2005), whether the translation rate of the viral-cellular fusion mRNAs also increases is unknown and deserves further experimental analysis. In Table 2 we summarize the diverse events of viral-cellular fusion transcripts reported in different cells or tissues that generate mRNAs possessing chimeric 3′-UTRs and the consequences in cancer.

**TABLE 2:** Transcription of chimeric virus-cell 3′-UTR upon the viral DNA integration into the host cells genome.

Virus	Cellular gene fussed with the viral E6,E7 mRNAs	Biological consequence on cancer	Sample source^a^	Integration site	Reference
HPV-16	Non-determined	Integration of HPV-16 DNA and expression of virus-cell mRNAs results in the increased stability of E6 and E7 mRNAs and cellular growth	W12 clonal populations and cervical tumor cell lines (SiHa, CaSki)	HPV integration does not occur at any specific locus on the host genome	Jeon and Lambert (1995)
HPV-16	*EIF1*, *MAP4*, *FAM110B*	Viral-cellular fusion transcripts are more stable than episome-derived transcripts, promoting carcinogenesis	Cervical carcinoma tumors	*EIF1*, *MAP4*, *FAM110B*	Ehrig et al. (2020)
HPV-16	*MTRF-1, HNRPA3, Rb, SGK1, MYC, Integrin α-2, FAP-1/PTPN13, HMG AT-hook, Fer tyrosine kinase, Plexin D1*	HPV integration is a random process that promotes selection of aggressively expanding cells. Some of those i9ntergation events may hit oncogenes with their concomitant deregulation	HNC tumors, human keratinocytes derived from primary tonsillar or foreskin epithelia immortalized	*MTRF-1, HNRPA3, Rb, SGK1, MYC, Integrin α-2, FAP-1/PTPN13, HMG AT-hook, Fer tyrosine kinase, Plexin D1*	Lace et al. (2011)
HPV-16, HPV-18	Non-determined	HPV-16/-18 DNA integration into host cell genome is related to the progression stage of vulvar dysplasia	VIN lesions and cervical carcinoma cell lines (SiHa and Caski)	Non-determined	Hillemanns and Wang (2006)
HPV-16, HPV-18, HPV-31, HPV-33, HPV-35	*CYS1, LRP1B, GPD2, LEPREL1, RREB1, MRV11, SSPN, NPM3, KLF7, COG8, RAD51L1, AGBL1, CFTR, EIF1, CEACAM6, MID1*	There is no correlation between HPV type and specific integration loci. HPV integration may occur in transcriptionally active regions and can influence on host gene expression	Ca, CIN3	*CYS1, LRP1B, GPD2, LEPREL1, RREB1, MRV11, SSPN, NPM3, KLF7, COG8, RAD51L1, AGBL1, CFTR, EIF1, CEACAM6, MID1*	Kraus et al. (2008)
HPV-2, HPV-6, HPV-11, HPV-13, HPV-16, HPV-18, HPV-30, HPV-31, HPV-35	Non-determined	The 3′ region HPV-16 mRNA represses the expression at both mRNA and protein level. For other HPV types, except 18, the 3′ region represses mRNA expression by posttranscriptional mechanisms	HaCaT and SiHa cells	Non-determined	Vinther et al. (2005)
HPV-16, HPV-18	N-*myc*	Integration of HPV genome results in overexpression of the oncogene c-*myc* and tumor progression	Invasive genital carcinomas	Chromosome band 8q24.1, nearby the protooncogene c-*myc*; chromosome band 2p24	Couturier et al. (1991)
HPV-16, HPV-18	Non-determined	The steady-state levels of c-*myc* mRNA are elevated in HeLa and C4-I cells relative to other Ca cell lines. Thus, This may spurr malignant transformation of cervical cells	Primary Ca and Ca cells (SiHa, SW756, HeLa, and C4-1)	Chromosome regions 20pter->20ql3 and 3p25->3qter, nearby the protooncogenes c-*src*-1 and c-*raf*-1; Chromosome region 13ql4-+13q32; Chromosome 12; Chromosome 8, 5′ of the c-*myc* gene	Durst et al. (1987)
HPV-16	Non-determined	Non-determined	Ca and normal placentas	No cellular sequence specific for the integration is found; some samples showed *Alu* repetitive sequences in the 5′- or 3′-flanking cellular sequences	Wagatsuma et al. (1990)
HPV-16	Non-determined	Non-determined	Keratinocyte SK-v cells; intraepithelial neoplasms termed bowenoid papulas	Non-determined	Schneider-Maunoury et al. (1987)

^a^ HNC, head and neck cancer; Ca, Cervical carcinoma; CIN, cervical intraepithelial neoplasia; VaIN, vaginal intraepithelial neoplasia; VIN, vulval intraepithelial neoplasia.

### Old questions, new insights

Cervical cancer remains a significant global public health challenge. Despite substantial advances in understanding the translation of HPV mRNAs, several longstanding questions remain unresolved. Among them is the mechanism by which the E5, L1, and L2 cistrons are translated. We speculate that these cistrons may utilize internal ribosome entry sites (IRESs); however, this hypothesis requires experimental validation. For certain viral proteins, such as E7 and E2, alternative translation mechanisms have been proposed. Therefore, further studies are necessary to clarify these observations. Additionally, the impact of cellular fragment fusion on the translation of chimeric virus–host mRNAs remains largely unexplored.

To date, most studies investigating the translation of HPV proteins have been conducted in heterologous systems—such as monkey COS-7 cells, mouse NIH3T3 cells, or rabbit reticulocyte lysates. Therefore, these findings must be validated in human cervical tissues, particularly throughout the process of epithelial differentiation during carcinogenesis.

Understanding the translation mechanisms of viral mRNAs is crucial for the future development of targeted therapies for cervical cancer. This will be instrumental in designing therapeutic strategies that combine translation inhibitors with agents directly targeting viral oncoproteins. Recent findings have highlighted promising pharmacological approaches. Two molecules targeting eIF4E, a key translation initiation factor, are currently being investigated for the treatment of HPV-associated cancers. Cercosporamide has been shown to reduce chemotherapy-induced phosphorylated eIF4E (p-eIF4E) levels, which are implicated in cancer progression, in mouse tumor models (Zhu et al., 2021). Ribavirin, another agent targeting p-eIF4E, has undergone clinical evaluation for HPV-related malignancies. Oral administration of ribavirin resulted in decreased p-eIF4E levels and was well-tolerated (Burman et al., 2022) (clinical trial NCT02308241; ClinicalTrials.gov).

Finally, several drugs are currently undergoing testing in animal models or clinical trials targeting the translation machinery across various cancers (García et al., 2024). These research efforts will be pivotal for the future development of cervical cancer therapies.

## References

[B1] Ajiro M. Zheng Z. M. (2015). E6^E7, a novel splice isoform protein of human papillomavirus 16, stabilizes viral E6 and E7 oncoproteins via HSP90 and GRP78. mBio 6, e02068–e02014. 10.1128/mBio.02068-14 25691589 PMC4337564

[B2] Alloul N. Sherman L. (1999). The E2 protein of human papillomavirus type 16 is translated from a variety of differentially spliced polycistroniic mRNAs. J. Gen. Virol. 80, 29–37. 10.1099/0022-1317-80-1-29 9934680

[B3] Alteri R. Baptiste D. Bell E. B. (2025). Cancer prevention and early detection. Facts and figures 2025-2026. American Cancer Society. 860025.

[B4] Basukala O. Banks L. (2021). The not-so-good, the bad and the ugly: HPV E5, E6 and E7 oncoproteins in the orchestration of carcinogenesis. Viruses 13, 1892. 10.3390/v13101892 34696321 PMC8541208

[B5] Bhattacharrjee R. Das S. S. Biswal S. S. Nath A. Das D. Basu A. (2021). Mechanistic role of HPV-associated early proteins in cervical cancer: molecular pathways and targeted therapeutic strategies. Crit. Rev. Oncol. Hematol. 174, 103675. 10.1016/j.critrevonc.2022.103675 35381343

[B6] Brant A. C. Menezes A. N. Felix S. P. de Almeida L. M. Sammeth M. Moreira M. A. M. (2018). Characterization of HPV integration, viral gene expression and E6E7 alternative transcripts by RNA-Seq: a descriptive study in invasive cervical cancer. Genomics 111, 1853–1861. 10.1016/j.ygeno.2018.12.008 30552977

[B7] Braunstein T. H. Madsen B. S. Gavnholt B. Rosenstierne M. W. Koefeld Johnsen C. Norrild B. (1999). Identification of a new promoter in the early region of the human papillomavirus type 16 genome. J. Gen. Virol. 80, 3241–3250. 10.1099/0022-1317-80-12-3241 10567657

[B8] Brito Querido J. Días-López I. Ramakrishnan V. (2024). The molecular basis of translation initiation and its regulation in eukaryotes. Nat. Rev. Mol. Cell. Biol. 25, 168–186. 10.1038/s41580-023-00624-9 38052923

[B9] Burman B. Drutman S. B. Fury M. G. Wong R. J. Katabi N. Ho A. L. (2022). Pharmacodynamic and therapeutic pilot studies of single-agent ribavirin in patients with human papillomavirus-related malignancies. Oral Oncol. 128, 105806. 10.1016/j.oraloncology.2022.105806 35339025 PMC9788648

[B10] Chan J. J. Tabatabaeian H. Tay Y. (2023). 3' UTR heterogeneity and cancer progression. Trends Cell Biol. 33, 568–582. 10.1016/j.tcb.2022.10.001 36372614

[B11] Chatterjee S. Pal J. P. (2009). Role of 5'- and 3'-untranslated regions of mRNAs in human diseases. Biol. Cell 101, 251–262. 10.1042/BC20080104 19275763

[B12] Cladel N. M. Bertotto A. Christensen N. D. (2010). Human alpha and beta papillomaviruses use different synonymous codon profiles. Virus Genes 40, 329–340. 10.1007/s11262-010-0451-1 20157772 PMC3752370

[B13] Collaborators G. V. C. (2025). Global, regional, and national trends in routine childhood vaccination coverage from 1980 to 2023 with forecasts to 2030: a systematic analysis for the global burden of disease study 2023. Lancet 406, 235–260. 10.1016/S0140-6736(25)01037-2 40578370 PMC12338332

[B14] Couturier J. Sastre-garau X. Schneider-Maunoury S. Labib A. Orth G. (1991). Integration of papillomavirus DNA near myc genes in genital carcinomas and its consequences for proto-oncogene expression. J. Virol. 65, 4534–4538. 10.1128/JVI.65.8.4534-4538.1991 1649348 PMC248900

[B15] De la Cruz-Hernández E. D. García-Carrancá A. Mohar-Betancourt A. Dueñas-González A. Contreras-paredes A. Pérez-Cárdenas E. (2005). Differential splicing of E6 within human papillomavirus type 18 variants and functional consequences. J. Gen. Virol. 86, 2459–2468. 10.1099/vir.0.80945-0 16099904

[B16] De Pasquale C. Kanduc D. (1998). Modulation of HPV16 E7 translation by tRNAs in eukaryotic cell-free translation systems. Biochem. Mol. Biol. Int. 45, 1005–1009. 10.1002/iub.7510450518 9739465

[B17] Della Fera A. N. Warburton A. Coursey T. L. Khurana S. Mcbride A. A. (2021). Persistent human papillomavirus infection. Viruses 13, 321. 10.3390/v13020321 33672465 PMC7923415

[B18] Doomer J. Van Doorslaer K. Afrasiabi C. Browne K. Ezejii S. Kim L. (2024). PaVE 2.0: behind the scenes of the papillomavirus episteme. J. Mol. Biol. 437, 168925. 10.1016/j.jmb.2024.168925 39732323 PMC12145264

[B19] Doorbar J. Parton A. Hartley K. Banks L. Crook T. Stanley M. (1990). Detection of novel splicing patterns in a HPV16-containing keratinocyte cell line. Virology 178, 254–262. 10.1016/0042-6822(90)90401-c 2167553

[B20] Doorbar J. Egawa N. Griffin H. Kranjek C. Mrakami I. (2015). Human papillomavirus molecular biology and disease association. Rev. Med. Virol. 25 (Suppl. 1), 2–23. 10.1002/rmv.1822 25752814 PMC5024016

[B21] Duensing S. Duensing A. Crum C. P. Munger K. (2001). Human papillomavirus type 16 E7 oncoprotein-induced abnormal centrosome synthesis is an early event in the evolving malignant phenotype. Cancer Res. 61, 2356–2360. 11289095

[B22] Durst M. Croce C. M. Gissmann L. Schwartz E. Huebner K. (1987). Papillomavirus sequences integrate near cellular oncogenes in some cervical carcinomas. Proc. Natl. Acad. Sci. U.S.A. 84, 1070–1074. 10.1073/pnas.84.4.1070 3029760 PMC304363

[B23] Durst M. Bosch F. X. Glitz D. Schneider A. zur Hausen H. (1991). Inverse relationship between human papillomavirus (HPV) type 16 early gene expression and cell differentiation in nude mouse epithelial cysts and tumors induced by HPV-positive human cell lines. J. Virol. 65, 796–804. 10.1128/JVI.65.2.796-804.1991 1846200 PMC239819

[B24] Ehrig F. Häfner N. Driesch C. Kraus Christiansen I. Beer K. Schmitz M. (2020). Differences in stability of viral and viral-cellular fusion transcripts in HPV-induced cervical cancers. Int. J. Mol. Sci. 211, 112. 10.3390/ijms21010112 31877944 PMC6981427

[B25] Elfakess R. Dikstein R. (2008). A translation initiation element specific to mRNAs with very short 5UTR that also regulates transcription. PLoS One 3, e3094. 10.1371/journal.pone.0003094 18769482 PMC2518114

[B26] Elfakess R. Sinvani H. Haimov O. Svitkin Y. Sonenberg N. Dikstein R. (2011). Unique translation initiation of mRNAs-containing TISU element. Nucleic Acid. Res. 39, 7598–7609. 10.1093/nar/gkr484 21705780 PMC3177215

[B27] Estêvão D. Costa N. R. Gil da Costa R. M. Medeiros R. (2019). Hallmarks of HPV carcinogenesis: the role of E6, E7 and E5 oncoproteins in cellular malignancy. Biochim. Biophys. Acta Gene Regul. Mech. 1862, 153–162. 10.1016/j.bbagrm.2019.01.001 30707946

[B28] Evande R. Rana A. Biswas-Fiss E. E. Biswas S. B. (2023). Protein-DNA interactions regulate human papillomavirus DNA replication, transcription, and oncogenesis. Int. J. Mol. Sci. 24, 8493. 10.3390/ijms24108493 37239839 PMC10218588

[B29] Félez-Sánchez M. Trosemeier J. H. Bedhomme S. González-Bravo M. I. Kamp C. Bravo I. G. (2015). Cancer, warts, or asymptomatic infections: clinical presentation matches codon usage preferences in human papillomaviruses. Genome Biol. Evol. 7, 2117–2135. 10.1093/gbe/evv129 26139833 PMC4558848

[B30] Gandin V. Masvidal L. Hulea L. Gravel S. Cargnello M. Mclaughlan S. (2016). nanoCAGE reveals 5′UTR features that define specific modes of translation of functionally related mTOR-sensitive mRNAs. Genome Res. 26, 636–648. 10.1101/gr.197566.115 26984228 PMC4864462

[B31] García A. Maldonado G. González J. L. Svitkin Y. Cantú D. García-Carrancá A. (2021). High-risk human papillomavirus-18 uses an mRNA sequence to synthesize oncoprotein E6 in tumors. Proc. Natl. Acad. Sci. U.S.A. 118, e2108359118. 10.1073/pnas.2108359118 34615711 PMC8522272

[B32] García A. Maldonado G. Hernández G. (2024). Translational control of papillomavirus mRNAs in the spotlight. Trends Cell Biol. 34, 703–706. 10.1016/j.tcb.2024.07.001 39069439

[B33] Glahder J. A. Hansen C. N. Vinther J. Madsen B. S. Norrild B. (2003). A promoter within the E6 ORF of human papillomavirus type 16 contributes to the expression of the E7 oncoprotein from a monocistronic mRNA. J. Gen. Virol. 84, 3429–3441. 10.1099/vir.0.19250-0 14645924

[B34] Graham S. V. (2010). Human papillomavirus: gene expression, regulation and prospects for novel diagnostic methods and antiviral therapies. Future Microbiol. 5, 1493–1506. 10.2217/fmb.10.107 21073310 PMC3527891

[B35] Graham S. V. (2017). Keratinocyte differentiation-dependent human papillomavirus gene regulation. Viruses 9, 245. 10.3390/v9090245 28867768 PMC5618011

[B36] Grassmann K. Rapp B. Maschek H. Petry K. U. Iftner T. (1996). Identification of a differentiation-inducible promoter in the E7 open reading frame of human papillomavirus type 16 (HPV-16) in raft cultures of a new cell line containing high copy numbers of episomal HPV-16 DNA. J. Virol. 70, 2339–2349. 10.1128/JVI.70.4.2339-2349.1996 8642661 PMC190076

[B37] Groves I. J. Coleman N. (2015). Pathogenesis of human papillomavirus-associated mucosal disease. J. Pathol. 235, 527–538. 10.1002/path.4496 25604863

[B38] Groves I. J. Coleman N. (2018). Human papillomavirus genome integration in squamous carcinogenesis: what have next-generation sequencing studies taught us? J. Pathol. 245, 9–18. 10.1002/path.5058 29443391

[B39] Gu W. Li M. Zhao W. M. Fang N. X. Bu S. Frazzer I. H. (2004). tRNASer(CGA) differentially regulates expression of wild-type and codon-modified papillomavirus L1 genes. Nucleic Acid. Res. 32, 4448–4461. 10.1093/nar/gkh748 15319446 PMC516046

[B40] Gu W. Ding J. Wang X. de Kluyver R. L. Saunders N. A. Frazer I. H. (2007). Generalized substitution of isoencoding codons shortens the duration of papillomavirus L1 protein expression in transiently gene-transfected keratinocytes due to cell differentiation. Nucleic Acid. Res. 35, 4820–4832. 10.1093/nar/gkm496 17621583 PMC1950544

[B41] Hernández G. Tettweiler G. (2012). “Protein abundance variation,” in Systems biology. Editor Meyer R. A. (Weinheim, Germany: John Wiley), 117–137.

[B42] Hernández G. Osnaya V. G. Pérez-Martínez X. (2019). Conservation and variability of the AUG initiation codon context in eukaryotes. Trends Biochem. Sci. 44, 1009–1021. 10.1016/j.tibs.2019.07.001 31353284

[B43] Hernández G. García A. Lasko P. Sonenberg N. (2020). Unorthodox mechanisms to initiate translation open novel paths for gene expression. J. Mol. Biol. 432, 166702. 10.1016/j.jmb.2020.10.035 33166539

[B44] Hershey J. W. B. Sonenberg N. Mathews M. B. (2019). Principles of translational control. Cold Spring Harb. Perspect. Biol. 11, a032607. 10.1101/cshperspect.a032607 29959195 PMC6719596

[B45] Hillemanns P. Wang X. (2006). Integration of HPV-16 and HPV-18 DNA in vulvar intraepithelial neoplasia. Gynecol. Oncol. 100, 276–282. 10.1016/j.ygyno.2005.10.003 16300821

[B46] Hinnebusch A. G. Dever T. E. Sonenberg N. (2004). “Mechanisms and regulation of protein synthesis initiation in eukaryotes,” in Protein synthesis and ribosome structure. Editors Nierhaus K. H. Wilson D. N. (Weinheim: Wiley-VCH Verlag GmbH and Co. KGaA), 241–322.

[B47] Huang D. Wang X. Huang Z. Liu Y. Liu X. Gin T. (2022). 3' untranslated regions of tumor suppressor genes evolved specific features to favor cancer resistance. Oncogene 41, 3278–3288. 10.1038/s41388-022-02343-5 35523946

[B48] Jackson R. J. Hellen C. U. Pestova T. V. (2012). Termination and post-terminationevents in eukaryotic translation. Adv. Protein Chem. Struct. Biol. 86, 45–93. 10.1016/B978-0-12-386497-0.00002-5 22243581

[B49] Jan E. Mohr I. Walsh D. (2016). A cap-to-tail guide to mRNA translation strategies in virus-infected cells. Annu. Rev. Virol. 3, 283–307. 10.1146/annurev-virology-100114-055014 27501262

[B50] Jeon S. Lambert P. F. (1995). Integration of human papillomavirus type 16 DNA into the human genome leads to increased stability of E6 and E7 mRNAs: implications for cervical carcinogenesis. Proc. Natl. Acad. Sci. U. S. A. 92, 1654–1658. 10.1073/pnas.92.5.1654 7878034 PMC42578

[B51] Jeon S. Allen-Hoffmann B. L. Lambert P. F. (1995). Integration of human papillomavirus type 16 into the human genome correlates with a selective growth advantage of cells. J. Virol. 69, 2989–2997. 10.1128/JVI.69.5.2989-2997.1995 7707525 PMC188998

[B52] Johnsen C. K. Stanley M. Norrild B. (1995). Analysis of human papillomavirus type 16 E5 oncogene expression *in vitro* and from bicistronic messenger RNAs. Intervirology 38, 339–345. 10.1159/000150461 8880384

[B53] Jones C. E. Danovaro-Holliday M. C. Mwinnyaa G. Gacic-Dobo M. Francis L. Grevendonk J. (2024). Routine vaccination coverage - Worldwide, 2023. MMWR Morb. Mortal. Wkly. Rep. 73, 978–984. 10.15585/mmwr.mm7343a4 39480752 PMC11527360

[B54] Kajitani N. Schwartz S. (2022). The role of RNA-binding proteins in the processing of mRNAs produced by carcinogenic papillomaviruses. Semin. Cancer Biol. 86, 482–496. 10.1016/j.semcancer.2022.02.014 35181475

[B55] Kaleem S. Dahal U. D’evi S. Kour B. Kour S. A. (2025). Codon usage evolution in viruses: implications for survival and pathogenicity. J. Mol. Evol. 10.1007/s00239-025-10263-7 40906273

[B56] Kraus I. Driesch C. Vinokurova S. Hovig E. A. S. von Knebe M. Doeberitz M. (2008). The majority of viral-cellular fusion transcripts in cervical carcinomas cotranscribe cellular sequences of known or predicted genes. Cancer Res. 68, 2514–2522. 10.1158/0008-5472.CAN-07-2776 18381461

[B57] Kwan T. Thompson S. R. (2019). Noncanonical translation initiation in eukaryotes. Cold Spring Harb. Perspect. Biol. 11, a032672. 10.1101/cshperspect.a032672 29959190 PMC6442200

[B58] Lace M. J. Anson J. R. Klussmann J. P. Wang D. H. Smith E. M. Haugen T. H. (2011). Human papillomavirus type 16 (HPV-16) genomes integrated in head and neck cancers and in HPV-16-immortalized human keratinocyte clones express chimeric virus-cell mRNAs similar to those found in cervical cancers. J. Virol. 85, 1645–1654. 10.1128/JVI.02093-10 21123375 PMC3028875

[B59] LaVigne A. W. Triedman S. A. Randall T. C. Trimble E. L. Viswanathan A. N. (2017). Cervical cancer in low and middle income countries: addressing barriers to radiotherapy delivery. Gynecol. Oncol. Rep. 22, 16–20. 10.1016/j.gore.2017.08.004 28948205 PMC5602511

[B60] Leppek K. Das R. Barna M. (2018). Functional 5' UTR mRNA structures in eukaryotic translation regulation and how to find them. Nat. Rev. Mol. Cell Biol. 19, 158–174. 10.1038/nrm.2017.103 29165424 PMC5820134

[B61] Li J. Lu X. (2013). The emerging roles of 3' untranslated regions in cancer. Cancer Lett. 337, 22–25. 10.1016/j.canlet.2013.05.034 23726838

[B62] Lin C. T. Tsai Y. C. He L. Calizo R. Chou H. H. Chang T. C. (2006). A DNA vaccine encoding a codon-optimized human papillomavirus type 16 E6 gene enhances CTL response and anti-tumor activity. J. Biomed. Sci. 13, 481–488. 10.1007/s11373-006-9086-6 16649071

[B63] Markowitz L. Schiller J. T. (2021). Human papillomavirus vaccines. J. Infect. Dis. 224, S367–S378. 10.1093/infdis/jiaa621 34590141 PMC8577198

[B64] Martinez-Zapien D. Ruiz F. X. Poirson J. Mitschler A. Ramirez J. Forster A. (2016). Structure of the E6/E6AP/p53 complex required for HPV-mediated degradation of p53. Nature 539, 541–545. 10.1038/nature16481 26789255 PMC4853763

[B65] Mayr C. (2017). Regulation by 3′-untranslated regions. Annu. Rev. Genet. 51, 171–194. 10.1146/annurev-genet-120116-024704 28853924

[B66] Mesplede T. Gagnon D. Bergeron-Labrecque F. Azar I. Senechal H. Coutlée F. (2012). p53 degradation activity, expression, and subcellular localization of E6 proteins from 29 human papillomavirus genotypes. J. Virol. 86, 94–107. 10.1128/JVI.00751-11 22013048 PMC3255875

[B67] Moral-Hernández O. López-Urrutia E. Bonilla-Moreno R. Martínez-Salazar M. Arechaga-Ocampo E. Berumen J. (2010). The HPV-16 E7 oncoprotein is expressed mainly from the unspliced E6/E7 transcript in cervical carcinoma C33-A cells. Arch. Virol. 155, 1959–1970. 10.1007/s00705-010-0787-9 20865289

[B68] Mordstein C. Cano L. Morales A. C. Young B. Ho A. T. Rice A. M. (2021). Transcription, mRNA export, and immune evasion shape the codon usage of viruses. Genome Biol. Evol. 13, evab106. 10.1093/gbe/evab106 33988683 PMC8410142

[B69] Müller M. (2005). Codon optimization of papillomavirus genes. Methods Mol. Med. 119, 433–444. 10.1385/1-59259-982-6:433 16350416

[B70] National Cancer Institute (2021). Cervical cancer causes, risk factors, and prevention. Washington: National Cancer Institute.

[B71] Nelson C. W. Mirabello L. (2023). Human papillomavirus genomics: understanding carcinogenicity. Tumour Virus Res. 15, 200258. 10.1016/j.tvr.2023.200258 36812987 PMC10063409

[B72] Obanya D. I. Wootton L. M. Morgan E. L. (2025). Advances in understanding the mechanisms of the human papillomavirus oncoproteins. Biochem. Soc. Trans. 53, 565–577. 10.1042/BST20253041 40380881 PMC12224896

[B73] Olmedo-Nieva L. Munoz-Bello J. O. Contreras-Paredes A. Lizano M. (2018). The role of E6 spliced isoforms (E6*) in human papillomavirus-induced carcinogenesis. Viruses 10, 45. 10.3390/v10010045 29346309 PMC5795458

[B74] Panczyszyn A. Boniewska-Bernacka E. Glab G. (2018). Telomers and telomerase during human papillomavirus-induced papillomavirus. Mol. Diagn. Ther. 22, 421–430. 10.1007/s40291-018-0336-x 29777397 PMC6061425

[B75] Parvathy S. T. Udayasuriyan V. Bhadana V. (2022). Codon usage bias. Mol. Biol. Rep. 49, 539–565. 10.1007/s11033-021-06749-4 34822069 PMC8613526

[B76] Pavelescu L. A. Mititelu-Zafiu N. L. Mindru D. E. Vladareanu R. Curici A. (2025). Molecular insights into HPV-driven cervical cancer: oncoproteins, immune evasion, and epigenetic modifications. Microorganisms 13, 1000. 10.3390/microorganisms13051000 40431173 PMC12113743

[B77] Pelletier J. Sonenberg N. (2019). The organizing principles of eukaryotic ribosome recruitment. Annu. Rev. Biochem. 88, 307–335. 10.1146/annurev-biochem-013118-111042 31220979

[B78] Remm M. Remm A. Ustav M. (1999). Human papillomavirus type 18 E1 protein is translated from polycistronic mRNA by a discontinuous scanning mechanism. J. Virol. 73, 3062–3070. 10.1128/JVI.73.4.3062-3070.1999 10074156 PMC104066

[B79] Ren J. Lii Q. Shen W. Tan X. (2025). Decoding codon usage patterns in high-risk human papillomavirus genomes: a comprehensive analysis. Curr. Microbiol. 82, 148. 10.1007/s00284-025-04131-2 39987223

[B80] Romanczuk H. Thierry F. Howley P. M. (1990). Mutational analysis of cis elements involved in E2 modulation of human papillomavirus type 16 P97 and type 18 P105 promoters. J. Virol. 64, 2849–2859. 10.1128/JVI.64.6.2849-2859.1990 2159546 PMC249467

[B81] Rosendo-Chalma P. Antonio-Vejar V. Tejedor J. G. O. Segarra J. O. Crespo B. V. Bigoni-Ordoñez G. D. (2024). The hallmarks of cervical cancer: molecular mechanisms induced by human papillomavirus. Biology 13, 77. 10.3390/biology13020077 38392296 PMC10886769

[B82] Rosenstierne M. W. Vinther J. Hansen C. N. Prydsoe M. Norrild B. (2003). Identification and characterization of a cluster of transcription start sites located in the E6 ORF of human papillomavirus type 16. J. Gen. Virol. 84, 2909–2920. 10.1099/vir.0.19332-0 14573795

[B83] Ryczek N. Łyś A. Makałowska I. (2023). The functional meaning of 5'UTR in protein-coding genes. Int. J. Mol. Sci. 24, 2976. 10.3390/ijms24032976 36769304 PMC9917990

[B84] Samorski R. Gissmann L. Osen W. (2006). Codon optimized expression of HPV 16 E6 renders target cells susceptible to E6-specific CTL recognition. Immunol. Lett. 107, 41–49. 10.1016/j.imlet.2006.07.003 16949679

[B85] Schneider-Gädicke A. Schwarz E. (1986). Different human cervical carcinoma cell lines show similar transcription patterns of human papillomavirus type 18 early genes. EMBO J. 5, 2285–2292. 10.1002/j.1460-2075.1986.tb04496.x 3023067 PMC1167112

[B86] Schneider-Maunoury S. Croissant O. Orth G. (1987). Integration of human papillomavirus type 16 DNA sequences: a possible early event in the progression of genital tumors. J. Virol. 61, 3295–3298. 10.1128/JVI.61.10.3295-3298.1987 3041049 PMC255912

[B87] Schuster S. L. Hsieh A. C. (2019). The untranslated regions of mRNAs in cancer. Trends Cancer 5, 245–262. 10.1016/j.trecan.2019.02.011 30961831 PMC6465068

[B88] Sherman L. Alloul N. (1992). Human papillomavirus type 16 expresses a variety of alternatively spliced mRNAs putatively encoding the E2 protein. Virology 191, 953–959. 10.1016/0042-6822(92)90271-p 1333130

[B89] Sherman L. Alloul N. Golan I. Durst M. Baram A. (1992). Expression and splicing patterns of human papillomavirus type-16 mRNAs in pre-cancerous lesions and carcinomas of the cervix, in human keratinocytes immortalized by HPV 16, and in cell lines established from cervical cancers. Int. J. Cancer 50, 356–364. 10.1002/ijc.2910500305 1310488

[B90] Silanes I. L. Quezada M. P. Esteller M. (2007). Aberrant regulation of messenger RNA 3'-untranslated region in human cancer. Cell Oncol. 29, 1–17. 10.1155/2007/586139 17429137 PMC4618221

[B91] Simón D. Cristina J. Musto H. (2021). Nucleotide composition and codon usage across viruses and their respective hosts. Front. Microbiol. 12, 646300. 10.3389/fmicb.2021.646300 34262534 PMC8274242

[B92] Smotkin D. Wettstein F. O. (1986). Transcription of human papillomavirus type 16 early genes in a cervical cancer and a cancer-derived cell line and identification of the E7 protein. Proc. Natl. Acad. Sci. U. S. A. 83, 4680–4684. 10.1073/pnas.83.13.4680 3014503 PMC323805

[B93] Smotkin D. Prokoph H. Wettstein F. O. (1989). Oncogenic and nononcogenic human genital papillomaviruses generate the E7 mRNA by different mechanisms. J. Virol. 63, 1441–1447. 10.1128/JVI.63.3.1441-1447.1989 2536845 PMC247848

[B94] Stacey S. N. Jordan D. Snijders P. J. Mackett M. Walboomers J. M. Arrand J. R. (1995). Translation of the human papillomavirus type 16 E7 oncoprotein from bicistronic mRNA is independent of splicing events within the E6 open reading frame. J. Virol. 69, 7023–7031. 10.1128/JVI.69.11.7023-7031.1995 7474122 PMC189622

[B95] Stacey S. N. Jordan D. Williamson A. J. K. Brown M. Coote J. H. Arrand J. R. (2000). Leaky scanning is the predominant mechanism for translation of human papillomavirus type 16 E7 oncoprotein from E6/E7 bicistronic mRNA. J. Virol. 74, 7284–7297. 10.1128/jvi.74.16.7284-7297.2000 10906182 PMC112249

[B96] Steinberg T. Ohlschlager P. Sehr P. Osen W. Gissmann L. (2005). Modification of HPV 16 E7 genes: correlation between the level of protein expression and CTL response after immunization of C57BL/6 mice. Vaccine 23, 1149–1157. 10.1016/j.vaccine.2004.08.027 15629358

[B97] Stoler M. H. Rhodes C. R. Whitbeck A. Wolinsky S. M. Chow I. T. Broker T. R. (1992). Human papillomavirus type 16 and 18 gene expression in cervical neoplasias. Hum. Pathol. 23, 117–128. 10.1016/0046-8177(92)90232-r 1310950

[B98] Sung H. Ferlay J. Siegel R. L. Laversanne M. Soerjomataram I. Jemal A. (2021). Global cancer statistics 2020: GLOBOCAN estimates of incidence and mortality worldwide for 36 cancers in 185 countries. CA Cancer J. Clin. 71, 209–249. 10.3322/caac.21660 33538338

[B99] Tan T. M. C. Gloss B. Bernard H. U. Ting R. C. Y. (1994). Mechanism of translation of the bicistronic mRNA encoding human papillomavirus type 16 E6-E7 genes. J. Gen. Virol. 75, 2663–2670. 10.1099/0022-1317-75-10-2663 7931152

[B100] Tang S. Tao M. McCoy J. P. Zheng Z. M. (2006). The E7 oncoprotein is translated from spliced E6*I transcripts in high-risk human papillomavirus type 16- or type 18-positive cervical cancer cell lines via translation reinitiation. J. Virol. 80, 4249–4263. 10.1128/JVI.80.9.4249-4263.2006 16611884 PMC1472016

[B101] Thierry F. Heard J. M. Dartmann K. Yaniv M. (1987). Characterization of a transcriptional promoter of human papillomavirus 18 and modulation of its expression by simian virus 40 and adenovirus early antigens. J. Virol. 61, 134–142. 10.1128/JVI.61.1.134-142.1987 3023691 PMC255220

[B102] Toots M. Mannik A. Kivi G. Ustav M. Ustav E. Ustav M. (2014). The transcription map of human papillomavirus type 18 during genome replication in U2OS cells. PLoS One 9, e116151. 10.1371/journal.pone.0116151 25548925 PMC4280167

[B103] Tsakogiiannis D. Nikolaidis M. Zagourii F. Zografos E. Kottaridi C. al e. (2022). Mutation profile of HPV16 L1 and L2 genes in different geographic areas. Viruses 15, 141. 10.3390/v15010141 36680181 PMC9867070

[B104] Van Doorslaer K. Li Z. Xirasagar S. Maes P. Kaminsky D. Liiou D. (2017). The papillomavirus episteme: a major update to the papillomavirus sequence database. Nucleic Acid. Res. 45, D499–D506. 10.1093/nar/gkw879 28053164 PMC5210616

[B105] Vinther J. Rosenstierne M. W. Kristiansen K. Norrild B. (2005). The 3' region of human papillomavirus type 16 early mRNAs decrease expression. BMC Infect. Dis. 5, 83. 10.1186/1471-2334-5-83 16225671 PMC1266366

[B106] Wagatsuma M. Hashimoto K. Matsukura T. (1990). Analysis of integrated human papillomavirus type 16 DNA in cervical cancers: amplification of viral sequences together with cellular flanking sequences. J. Virol. 64, 813–821. 10.1128/JVI.64.2.813-821.1990 2153245 PMC249176

[B107] Wang X. Meyers C. Wang H. K. Chow L. T. Zheng Z.M. (2011). Construction of a full transcription map of human papillomavirus type 18 during productive viral infection. J. Virol. 85, 8080–8092. 10.1128/JVI.00670-11 21680515 PMC3147953

[B108] Wang Y. Chen F. Qu W. Gong Y. Wang Y. Chen L. (2025). Alternative splicing in the genome of HPV and its regulation. Front. Cell. Infect. Microbiol. 10.3389/fcimb.2024.1443868 39502170 PMC11534716

[B109] Wentzensen N. Ridder R. Klaes R. Vinukurova S. Schaefer U. Doeberitz M. v. K. (2002). Characterization of viral-cellular fusion transcripts in a large series of HPV16 and 18 positive anogenital lesions. Oncogene 21, 419–426. 10.1038/sj.onc.1205104 11821954

[B110] Wentzensen N. Vinokurova S. von Knebel Doeberitz M. (2004). Systematic review of genomic integration sites of human papillomavirus genomes in epithelial dysplasia and invasive cancer of the female lower genital tract. Cancer Res. 64, 3878–3884. 10.1158/0008-5472.CAN-04-0009 15172997

[B111] WHO (2022). International agency for research on cancer. Geneva: WHO. Available online at: https://www.iarc.who.int/cancer-type/cervical-cancer/900-world-fact-sheet.pdf (Accessed on September 15th, 2025).

[B112] Yu L. Majerciack V. Zheng Z.-M. (2022). HPV16 and HPV18 genome structure, expression, and post-transcriptional regulation. Int. J. Mol. Sci. 23, 4943. 10.3390/ijms23094943 35563334 PMC9105396

[B113] Zhang Y. Yang M. Yang S. Hong S. (2022). Role of noncoding RNAs and untranslated regions in cancer: a review. Medicine 101, e30045. 10.1097/MD.0000000000030045 35984196 PMC9388041

[B114] Zhao K. N. Chen J. (2011). Codon usage roles in human papillomavirus. Rev. Med. Virol. 21, 397–411. 10.1002/rmv.707 22025363

[B115] Zhao K. N. Liu W. J. Frazer I. H. (2003). Codon usage bias and A + T content variation in human papillomavirus genomes. Virus Res. 98, 95–104. 10.1016/j.virusres.2003.08.019 14659556

[B116] Zhao K. N. Gu W. Fang N. X. Saunders N. A. Frazer I. H. (2005). Gene codon composition determines differentiation-dependent expression of a viral capsid gene in keratinocytes *in vitro* and *in vivo* . Mol. Cell Biol. 25, 8643–8655. 10.1128/MCB.25.19.8643-8655.2005 16166644 PMC1265747

[B117] Zhou J. Liu W. J. Peng S. W. Sun X. Y. Frazer I. (1999). Papillomavirus capsid protein expression level depends on the match between codon usage and tRNA availability. J. Virol. 73, 4972–4982. 10.1128/JVI.73.6.4972-4982.1999 10233959 PMC112541

[B118] Zhu Y. Wang C. Li M. Yang X. (2021). Targeting of MNK/eIF4E overcomes chemoresistance in cervical cancer. J. Pharm. Pharmacol. 73, 1418–1426. 10.1093/jpp/rgab094 34254647

[B119] Ziegert C. Wentzensen N. Vinokurova S. Kisseljov F. Einekel J. Hoeckel M. (2003). Comprehensive analysis of HPV ntegration locii in anogenital lessions conbining transcript and genome-based amplification techniques. Oncogene 22, 3984. 10.1038/sj.onc.1206629 12813471

